# EGFR Mutation Positive Stage IV Non-Small-Cell Lung Cancer: Treatment Beyond Progression

**DOI:** 10.3389/fonc.2014.00350

**Published:** 2014-12-08

**Authors:** Katrijn Van Assche, Liesbeth Ferdinande, Yolande Lievens, Katrien Vandecasteele, Veerle Surmont

**Affiliations:** ^1^Department of Respiratory Medicine, Ghent University Hospital, Ghent, Belgium; ^2^Department of Pathology, Ghent University Hospital, Ghent, Belgium; ^3^Department of Radiation Oncology, Ghent University Hospital, Ghent, Belgium

**Keywords:** advanced non-small-cell lung cancer, EGFR mutation, local progression, radiotherapy, tyrosine kinase inhibitor

## Abstract

Non-small-cell lung cancer (NSCLC) is the leading cause of death from cancer for both men and women. Chemotherapy is the mainstay of treatment in advanced disease, but is only marginally effective. In about 30% of patients with advanced NSCLC in East Asia and in 10–15% in Western countries, epidermal growth factor receptor (EGFR) mutations are found. In this population, first-line treatment with the tyrosine kinase inhibitors (TKIs) erlotinib, gefitinib, or afatinib is recommended. The treatment beyond progression is less well-defined. In this paper, we present three patients, EGFR mutation positive, with local progression after an initial treatment with TKI. These patients were treated with local radiotherapy. TKI was temporarily stopped and restarted after radiotherapy. We give an overview of the literature and discuss the different treatment options in case of progression after TKI: TKI continuation with or without chemotherapy, TKI continuation with local therapy, alternative dosing or switch to next-generation TKI or combination therapy. There are different options for treatment beyond progression in EGFR mutation positive metastatic NSCLC, but the optimal strategy is still to be defined. Further research on this topic is ongoing.

## Introduction: Case Reports

**Figure 1:**
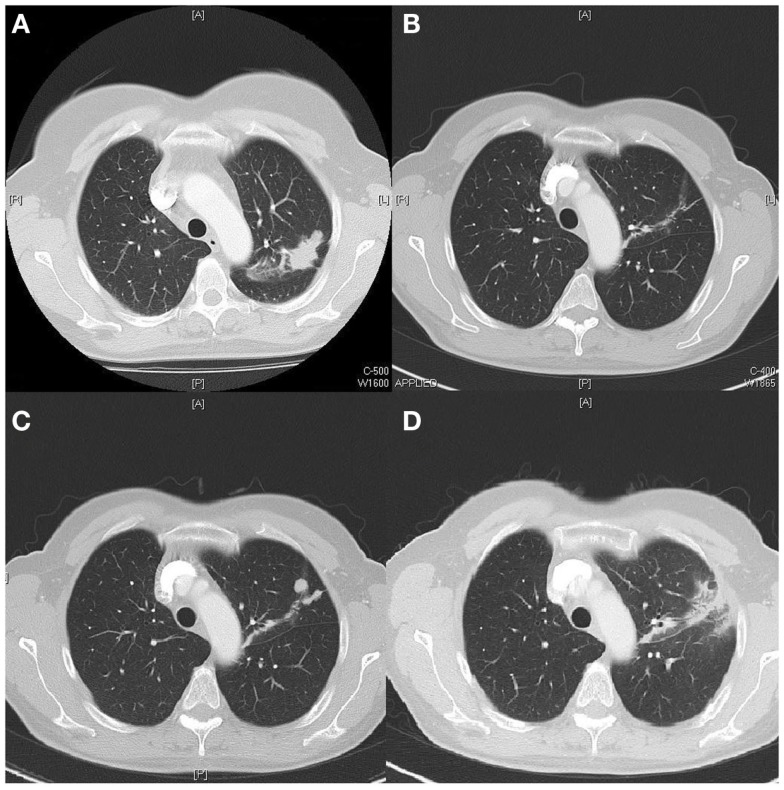
**(A)** Tumor upper left lobe on diagnosis (CT scan). **(B)** Near complete response 2 months after start TKI (CT scan). **(C)** Local progression 18 months after diagnosis (CT scan). **(D)** Partial response 3 months after local stereotactic radiotherapy (CT scan).

The first case concerns a 56 years old woman with a smoking history of 19 pack years (PY). In October 2011, she was diagnosed with a metastatic adenocarcinoma of the lung, epidermal growth factor receptor (EGFR) mutation positive with a deletion found on exon 19. The primary tumor was located in the left upper lobe (Figure 1A). There was a pericardial effusion that was proven to be metastatic.

In December 2011, the tyrosine kinase inhibitor (TKI) gefitinib (Iressa^®^) was started. The first follow up CT scan, 2 months after starting Iressa showed near complete response (Figure 1B).

In March 2013, 18 months after initial diagnosis, local progression is documented on the site of the primary tumor (Figure 1C) and staging by PET-CT showed no metastatic lesions. On the multidisciplinary oncology board, it was decided to give local stereotactic body radiotherapy (3 Gy × 20 Gy), which was started in April 2013. TKI treatment was temporarily stopped during radiotherapy. Three months after treatment a significant decrease of the tumor was seen (Figure 1D). Until October 2014, 17 months after completion of the radiotherapy, there is no evidence of disease and the patient continues TKI treatment.

**Figure 2:**
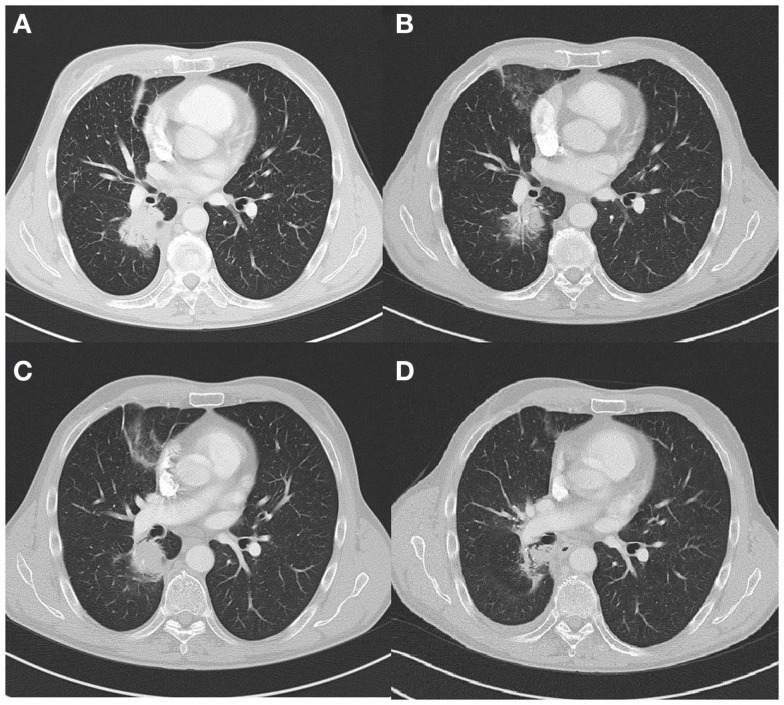
**(A)** Tumor lower right lobe on diagnosis (CT scan). **(B)** Partial response 22 months after start TKI (CT scan). **(C)** Local progression 26 months after diagnosis (CT scan). **(D)** Partial response 9 months after local radiotherapy (CT scan).

The second case concerns a 66 years old male ex-smoker (6 PY during adolescence). In May 2010, an EGFR mutation (L858R c.2573T > G) positive adenocarcinoma of the right lower lobe of the lung with metastasis in the contra-lateral lung was diagnosed (Figure 2A). In June 2010, afatinib, a irreversible EGFR-HER2-inhibitor, was started in a clinical trial (Gilotrif^®^ in BIBW 2992 trial). The patient experienced partial response (Figure 2B) until July 2012, 26 months after initial diagnosis. At that point, local progression was seen at the site of the primary tumor in the right upper lobe, and no distant metastases (Figure 2C). There was a multidisciplinary consensus to start local hypofractionated radiotherapy (20 Gy × 3 Gy) and stop TKI during this treatment. After completion of the radiotherapy gefitinib was started. A significant decrease of the tumor was seen (Figure 2D). One year later, in June 2013, a new pleural effusion was seen and proven to be metastatic disease. A switch to standard chemotherapy was initiated in October 2013 after pleurodesis. We note stable disease until April 2014. At that point, erlotinib was started because of progressive disease. Four months later, brain metastasis were diagnosed which where treated with whole brain radiotherapy (5 Gy × 4 Gy). Erlotinib was continued afterwards. Until October 2014, we note stable disease.

**Figure 3:**
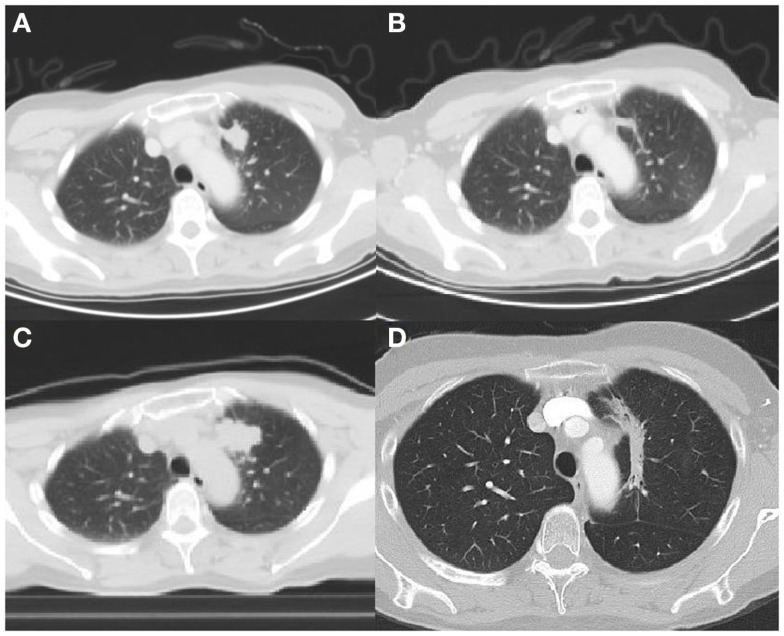
**(A)** Tumor left upper lobe on diagnosis (18F-FDG PET-CT scan). **(B)** Near complete response 32 months after start TKI (18F-FDG PET-CT scan). **(C)** Local progression 40 months after diagnosis (18F-FDG PET-CT scan). **(D)** Partial response 8 months after local radiotherapy (CT scan).

The third case concerns a 59 years old female never smoker. In January 2009, she was diagnosed with an adenocarcinoma of the lung (left upper lobe) with bone and liver metastasis (Figure 3A). The tumor was EGFR mutation positive, with a deletion found on exon 19. In February, erlotinib was started in a clinical trial (FIELT study) with near complete remission (Figure 3B). 18F-FDG PET-CT showed no distant metastases. After 34 months, the FIELT study was closed. At that point, in December 2011, we made a switch to gefitinib because erlotinib was not yet reimbursed in first-line treatment. In May 2012, 6 months after switch to gefitinib progression of the tumor in the left upper lobe was seen (Figure 3C) and local stereotactic body radiotherapy was given (8 Gy × 7.5 Gy). Gefinitib was stopped during radiotherapy but restarted afterwards. A significant decrease of the tumor was seen (Figure 3D).

Until October 2014, we note stable disease, 27 months after local therapy.

## Background

### EGFR mutation and tyrosine kinase inhibitors

Non-small-cell lung cancer (NSCLC) is the leading cause of death from cancer for both men and women. Chemotherapy is the mainstay of treatment in advanced disease, but is only marginally effective.

Epidermal growth factor inhibitors show promise in the treatment of metastatic NSCLC.

Responsiveness to these drugs is seen particularly in women, adenocarcinoma, non-smokers, and Asians. In the majority of these responding patients, a somatic mutation of the EGFR gene is found (1).

In 2004, EGFR mutations were described and characterized by high activity of EGFR-TKIs such as gefitinib and erlotinib. EGFR mutations are found in about 30% of patients with advanced NSCLC in East Asia and in 10–15% in Western countries (1–3).

**Figure 4:**
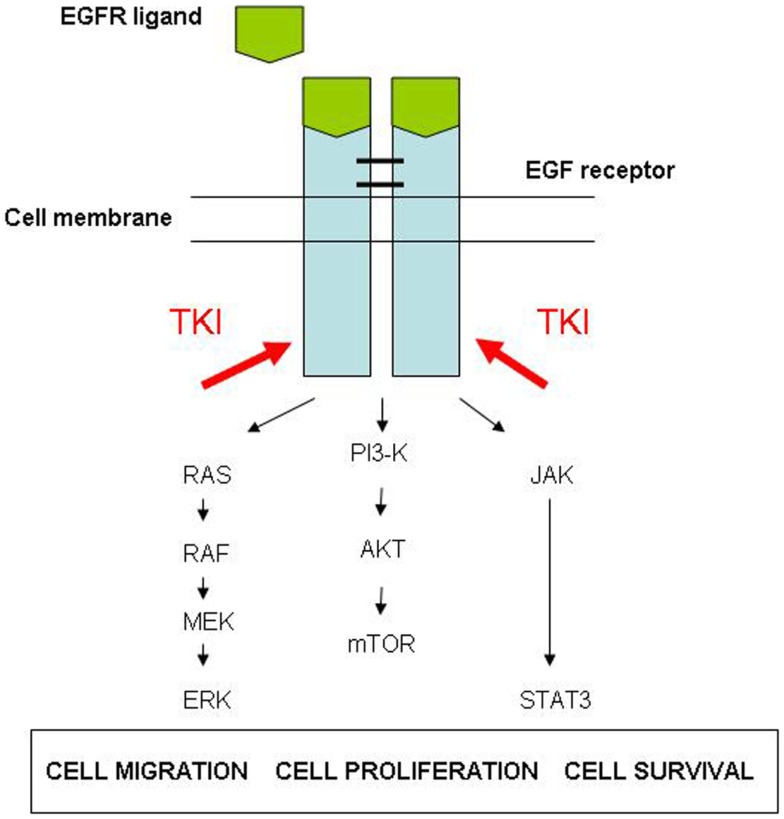
**Epidermal growth factor receptor pathway**.

Epidermal growth factor receptor is a transmembrane receptor. Its activation initiates a signal transduction cascade that promotes tumor-cell proliferation, survival, and migration (Figure 4). An activating mutation on the gene encoding for EGFR leads to an upregulation of the EGFR, which results in uncontrolled proliferation of tumor-cells, using the tyrosine kinase pathway.

Epidermal growth factor receptor or HER1 is a member of the HER-family, a transmembrane peptide receptor family, with tyrosine kinase activity. The HER-family comprises HER 1–4.

The TKI is a molecule that blocks binding of adenosine-5-triphosphate (ATP) to the tyrosine kinase catalytic domain. This interruption of the signaling pathway leads to massive apoptosis of mutant tumor-cells.

Common alterations on the gene encoding for EGFR are exon 19 deletions and the L858R point mutation in exon 21, covering >90% of all mutations and sensitive to TKI. The other EGFR mutations, e.g., the T790M mutation on exon 20, represent less than 10% of all mutations, and are associated with drug resistance (1).

### Treatment of patients with advanced NSCLC with EGFR mutation

Both the European Society of Medical Oncology (ESMO) (2) and the American College of Chest Physicians (ACCP) (3) recommend first-line treatment with a TKI (erlotinib or gefitinib) in metastatic NSCLC bearing an activating EGFR mutation because of higher response rate, longer progression free survival (PFS), and better quality of life when compared with first-line chemotherapy. These recommendations are based on five randomized phase III trials that compared the efficacy of EGFR-TKI vs. platinum based chemotherapy as first-line treatment (4–8) (Table 1). In these trials, there was no statistical significant survival advantage.

**Table 1:** **Phase III trials of EGFR tyrosine kinase inhibitors vs. chemotherapy as first-line treatment in patients with advanced NSCLC bearing EGFR mutation**.

Study	*n* (EGFR mutation positive patients)	EGFR-TKI	Chemotherapy	PFS median (months) TKI vs. chemotherapy	PFS hazard ratio (95% CI) TKI vs. chemotherapy
IPASS (4)	261	Gefitinib	Carboplatin + paclitaxel	9.5 vs. 6.3	0.48 (0.36–0.64)
NEJ002 (5)	224	Gefitinib	Carboplatin + paclitaxel	10.8 vs. 5.4	0.30 (0.22–0.41)
WJTOG3405 (6)	172	Gefitinib	Cisplatin + paclitaxel	9.2 vs. 6.3	0.49 (0.34–0.71)
OPTIMAL (7)	154	Erlotinib	Carboplatin + gemcitabine	13.1 vs. 4.6	0.16 (0.10–0.26)
EURTAC (8)	173	Erlotinib	Cisplatin (or carboplatin + docetaxel or gemcitabine	9.7 vs. 5.2	0.37 (0.25–0.54)
LUX-LUNG 3 (9)	345	Afatinib	Cisplatin + pemetrexed	11.1 vs. 6.9	0.58 (0.43–0.78)
LUX-LUNG 6 (10)	364	Afatinib	Cisplatin + gemcitabine	11 vs. 5.6	0.28 (0.20–0.39)

*PFS, progression free survival; EGFR, epidermal growth factor receptor; NSCLC, non-small cell lung cancer; TKI, tyrosine kinase inhibitor; CI, confidence interval*.

Also, afatinib, an irreversible TKI, showed a longer PFS in comparison with first-line chemotherapy in first-line treatment for EGFR mutation positive NSCLC (9, 10) (Table 1). A pooled analysis of two large open label phase III studies comparing afatinib with chemotherapy showed significant improvement of overall survival (OS) in patients with EGFR deletion 19. There was no significant difference in OS for patients with L858R mutations (11).

The most common adverse events associated with EGFR inhibitors are dermatologic toxicities, occurring in >50% of the patients. A papulopustular rash is the most frequently reported cutaneous side effect. Other reported cutaneous side effects are xerosis, telangiectasia, fissures, hyperpigmentation, and hair and nail changes. The papulopustular rash seems to be dose dependent, with higher-grade of skin toxicity at higher dose levels. The reaction is reversible, with usually a complete resolution within 4 weeks after ending treatment. In numerous studies, a correlation between skin rash severity and response to EGFR-TKI treatment was seen.

The incidence of diarrhea varies from 27 to 87% in phase III clinical trials. Severe diarrhea can lead to dehydration, electrolyte disturbances, and renal insufficiency.

Hepatitis or liver failure is rarely seen, but intermittent testing of liver function is recommended (12, 13).

In general, EGFR-TKIs are better tolerated than chemotherapy. This was documented in the quality of life analysis of the IPASS, OPTIMAL, and LUX-LUNG 3–6 trial (4, 7, 9, 10).

### EGFR mutation testing

European Society of Medical Oncology guidelines recommend routine EGFR mutation testing in all non-squamous tumors in patients with advanced or recurrent disease (14). EGFR testing may be performed in selected cases of squamous tumors, guided by clinical criteria (e.g., young age, minimal, or remote smoking) and especially in the setting of more limited lung cancer specimens (biopsy, cytology) in which an adenocarcinoma component cannot be completely excluded (14, 15). The role of the pathologist is not restricted to making a histological diagnosis, with prudent use of immunohistochemistry in morphologically undifferentiated cases of NSCLC, but he or she should also be actively involved in sample reviewing, selection, and preparation for DNA extraction for EGFR mutation testing. Mostly, formalin-fixed, paraffin-embedded (FFPE) tissue is used, but also cytologic samples (and particularly cell blocks), which account for up to 40% of all NSCLC diagnoses, are suitable for EGFR mutation analysis (14, 15). According to the CAP/IASCL/AMP molecular testing guidelines, EGFR mutation tests should be able to detect mutations in specimens with at least 50%, but ideally 10% tumor-cell content. PCR-based methods are often preferred as they offer efficient and sensitive assays allowing adequate internal and external validation (15). ESMO and CAP/IASCL/AMP guidelines recommend analysis of a wide coverage of mutations in exon 18–21, including those associated with therapy resistance (14). Quality control of these EGFR mutation tests is mandatory and laboratories performing these assays should enroll in external quality assurance programs on a regular basis (14, 15). The pathology department of our center used the Therascreen EGFR RGQ PCR kit (Qiagen) to determine EGFR mutation status in the cases described and obtained ISO15189 accreditation for this test since May 2012.

As the number of biomarkers to be tested for lung cancer and the number of targeted therapies are expected to increase in the near future, new strategies are under development to allow testing for multiple biomarkers on limited specimen volumes instead of the single-target-gene approach of most traditional assays. Multiplex technologies such as next-generation sequencing are rapidly evolving as a molecular diagnostic tool to meet these requirements and are already implemented in daily routine practice (16).

### Intrinsic and acquired resistance to a TKI

There are two types of resistance: primary or intrinsic resistance, occurring between the first and third month of treatment, and secondary resistance or acquired resistance occurring later, after an initial response to EGFR-TKI. Jackman et al. (17) defined secondary resistance with the following criteria: previous treatment with a single-agent EGFR-TKI; either both of the following: a tumor that harbors an EGFR mutation known to be associated with drug sensitivity or objective clinical benefit from treatment with an EGFR-TKI; systemic progression of disease (RECIST or WHO) while on continuous treatment with gefitinib or erlotinib within the last 30 days; and no intervening systemic therapy between cessation of EGFR-TKI and initiation of new therapy (Table 2).

**Table 2:** **Criteria for acquired resistance to EGFR-TKI in lung cancer**.

1. Prior therapy with a EGFR-TKI (monotherapy)
2. One of the two following
- Tumor with an EGFR mutation known to be associated with drug sensitivity (e.g., exon 19 deletion, L858R, G719X)
- A documented partial or complete response or a significant and prolonged stable disease, based on the RECIST or WHO criteria (18, 19), after treatment with a EGFR-TKI
3. Disease progression while on continuous treatment with EGFR-TKI during the last 30 days
4. No additional systemic therapy since discontinuation of EGFR-TKI

*EGFR, epidermal growth factor receptor; TKI, tyrosine kinase inhibitor; RECIST, response evaluation criteria in solid tumors (18); WHO, World Health Organization (19)*.

The etiology of primary resistance is not well-known. Possible mechanisms are a false-positive test result for EGFR mutation, resistant mutations or other downstream or parallel molecular abnormalities in the EGFR signaling pathway or mixed tumors containing small cell components on initial diagnosis. These resistant mutations or other downstream or parallel molecular abnormalities are similar to the mechanisms of acquired resistance.

The mechanisms of acquired resistance can be divided in three categories:

#### Genetic alterations in EGFR: secondary mutations and target gene amplification

In approximately 50% of TKI-resistant, EGFR-mutant patients a T790M mutation was found, which is a secondary mutation in exon 20 of EGFR. The resistance is caused predominantly through changes in ATP affinity. In EGFR-mutant tumors, the affinity for ATP is reduced, but the addition of T790M leads to re-establishing ATP as favored substrate rather than the TKI. Three, less frequent, secondary EGFR mutations are described: D761Y, T854A, and L747S.

Another cause of acquired resistance is EGFR gene amplification, which may shift the intracellular balance between kinase and TKI in favor of the kinase (20).

#### Bypass signaling

A pathway that bypasses the inhibited EGFR can also cause TKI resistance. One well-described example is MET-amplification, with an estimated frequency of 5–22% of TKI-resistant, EGFR-mutant patients. MET-amplified subclones preexist in untreated tumor cells, but can cause resistance as MET-amplification occurs as a result of selective pressure. Activation of MET through its ligand, hepatocyte growth factor (HGF), may also promote resistance. Other known bypass signaling tracts are HER-2 amplification, BRAF mutations, and PIK3CA mutations (20).

#### Phenotypic alterations

Several reports describe transformations of EGFR-mutant NSCLC to small-cell lung cancer (SCLC) or high-grade neuroendocrine carcinoma.

The underlying mechanism is not known (20).

#### Other considerations

The combination of different resistance mechanisms is possible within the same biopsy specimen or in specimen from different tumor localizations within the same patient. This can lead to disease “flare” on TKI discontinuation, because the pressure on sensitive tumor cells is removed.

We also have to take in account the pharmacokinetic mechanisms (e.g., influence of smoking, proton pump inhibitor intake, concomitant food intake, concomitant use of CYP3A4 inhibitors or inductors) and bad compliance, when intrinsic or acquired resistance is suspected (20).

## Discussion: Treatment Beyond Progression

This topic is still a subject of discussion. The ESMO Guidelines state that continuation treatment beyond progression is “an issue remaining to be defined” (2). In the evidence-based clinical practice guidelines of the ACCP the second and third-line treatment after first-line TKI for metastatic NSCLC bearing EGFR mutation is not discussed (3).

The NCCN Clinical Practice Guidelines of 2013 added an algorithm for treatment of patients who progress on erlotinib. They suggest that erlotinib can be continued in case of asymptomatic progression, brain metastasis, or local progression. However, additional therapy may be added (whole brain radiotherapy, systemic therapy, local therapy) (21).

In this literature different approaches for treatment beyond progression are discussed.

### TKI continuation with or without chemotherapy

Subsequent chemotherapy in patients with EGFR-TKI failure has been proven to give significant longer OS and PFS than best supportive care (22).

But should TKI be maintained and combined with chemotherapy?

The results of a review of Leung and Mok showed no improvement of treatment outcome in EGFR mutation-positive patients, treated with concurrent combination of chemotherapy and EGFR-TKI in first-line (23). Since TKI induces a G1-phase cell-cycle arrest, cell-cycle phase-dependent chemotherapeutic agents will not be effective during that arrest (24).

In the FASTACT II trial, a phase III trial, the sequential intercalated combination of chemotherapy and EGFR-TKI showed improvement of PFS in first-line treatment of stage IIIb/IV NSCLC adenocarcinoma bearing the EGFR mutation (25). If the combination of chemotherapy and TKI in an sequential intercalated schedule can improve outcome beyond progression is still a topic of research.

The LUX-Lung 5, a randomized, open label, phase III trial compared afatinib plus paclitaxel to investigator’s choice chemotherapy in patients with metastatic NSCLC progressed on erlotinib/gefitinib and afatinib. The combination showed improved PFS and objective response rate (ORR) vs. chemotherapy alone (26).

The IMPRESS trial, a randomized phase III trial evaluating the addition of gefitinib to cisplatin/pemetrexed as second-line treatment after gefitinib failure, showed no clinical benefit for the combination therapy in comparison with doublet chemotherapy alone (27).

Another possible therapeutic option is to continue TKI without chemotherapy. This option is mostly used in patients with slowly or oligo-metastatic disease. For the latter the combination with a local therapy is preferable.

### TKI continuation with local therapy

A retrospective cohort study (28) with 18 patients showed that local therapy [surgery, radiation therapy, radiofrequency ablation (RFA)] could be a useful option in case of local progression. In the evaluated patients, local therapy in combination with continuation of TKI (restarted TKI within 1 month of local therapy) led to a long PFS (median time 10 months) and OS (median time 41 months). Most patients had surgery and only a minority had radiation therapy or RFA. Similar results were seen in a small retrospective study with nine patients (29), who received radiotherapy in combination with continuation of TKI, for local disease progression.

Also, for this approach further research is ongoing (Table 3).

**Table 3:** **Overview ongoing trials**.

Identifying number (status)	Phase	Description
**TKI ± CHEMOTHERAPY**		
NCT01746277 (recruiting)	II	Chemotherapy (docetaxel or pemetrexed) sequenced by or combined with gefitinib after progression
NCT01928160 (not yet recruiting)	II	Chemotherapy (pemetrexed and carboplatin or cisplatin) with or without erlotinib hydrochloride in treating patient with stage IV non-small cell lung cancer resistant to first-line therapy with erlotinib hydrochloride or gefitinib
NCT02098954 (not yet recruiting)	II	Erlotinib combined with chemotherapy (gemcitabine) in TKI-resistant non-small cell lung cancers
NCT01998061 (recruiting)	II	Continuation of TKI with or without chemotherapy beyond gradual progression
NCT02064491 (recruiting)	II	Erlotinib treatment with or without chemotherapy beyond progression in EGFR-mutant NSCLC
**LOCAL THERAPY**		
NCT01573702 (recruiting)	II	Stereotactic radiosurgery or other local ablation followed by erlotinib for patients with epidermal growth factor receptor (EGFR) mutation who have previously progressed on an epidermal growth factor receptor-tyrosine kinase inhibitor (EGFR-TKI)
**ALTERNATIVE DOSING OR SWITCH TO NEXT-GENERATION TKI**		
NCT01530334 (ongoing)	II	Iressa re-challenge in advanced NSCLC EGFR M+ patients who responded to gefitinib used as 1st line or previous treatment (ICARUS)
NCT01932229 (recruiting)	II	An open label study of BIBW 2992/afatinib in advanced non-small cell lung cancer patients pre-treated with erlotinib or gefitinib
NCT01526928 (recruiting)	I/II	CO-1686 (third-generation TKI) in second or third-line treatment for EGFR positive NSCLC, with disease progression under first or second-generation TKI
**COMBINATION THERAPY**		
NCT01982955 (recruiting)	I/II	MSC2156119J in combination with gefitinib in subjects with MET positive locally advanced or metastatic non-small cell lung cancer (NSCLC) harboring epidermal growth factor receptor (EGFR) mutation and having acquired resistance to first-line gefitinib
NCT01610336 (recruiting)	I/II	INC280 administered orally in combination with gefitinib in adult patients with EGFR mutated, c-MET-amplified non-small cell lung cancer who have progressed after EGFR inhibitor treatment
NCT01900652 (recruiting)	II	A study of LY2875358 in non-small cell lung cancer participants
NCT01090011 (ongoing)	I	BIBW 2992 (afatinib) plus cetuximab (Erbitux^®^) in patients with non-small cell lung cancer with progression following prior erlotinib (Tarceva^®^) or gefitinib (Iressa^®^)
NCT01259089 (ongoing)	I/II	Hsp90 inhibitor AUY922 and erlotinib hydrochloride in treating patients with stage IIIB-IV non-small cell lung cancer
NCT01646125 (recruiting)	II	An open label, randomized phase II study to evaluate the efficacy of AUY922 vs. pemetrexed or docetaxel in NSCLC patients with EGFR mutations

### Alternative dosing or switch to next-generation TKI

Increasing TKI dosage or switching to another TKI has been applied in several series of patients and case reports (30, 31). In general, EGFR-TKIs have a low capacity to penetrate into the cerebral fluid, although erlotinib achieved a relatively higher level of CNS penetration. Switching from gefitinib to erlotinib in case of brain or meningeal progression was applied in a small retrospective series of seven patients. Three patients showed partial response, three had stable disease, and one had progressive disease. Performance status and symptoms improved in five patients. The OS from the initiation of erlotinib treatment ranged from 15 to 530 days (median, 88 days) (32). This was not yet confirmed by larger prospective randomized trials.

Also, increasing the dosage can be beneficial in case of brain or meningeal progression. One retrospective series showed a partial response on central nervous system (CNS) radiography in six out of nine patients after treating with high-dose erlotinib (1500 mg once weekly), one patient had stable disease, and two had progressive disease. The median time to CNS progression was 2.7 months (range 0.4–14.5 months) and the median OS was 12 months (range 2.5 – not reached). Further prospective randomized trials are necessary to confirm this approach (33).

If progression is linked to acquired resistance, no benefit can be expected from increasing dosage or switching to another first-generation TKI.

In contrast with erlotinib and gefinitib, second-generation EGFR-TKIs, such as afatinib, dacomitinib, and neratinib, bind irreversible with EGFR. They also possess activity against other HER-family members, like HER2. In the LUX-lung 1 trial, afatinib had a significant longer PFS in comparison with placebo, but showed no improvement of OS. Furthermore, the trial showed an important drug-related toxicity in the afatinib-group (34). For neratinib, a phase II trial showed disappointing results (35) and the dacomitinib data showed possible benefit in patients with erlotinib resistance (36), but further investigations are necessary.

In preclinical studies, third-generation EGFR inhibitors (WZ4002 and CO-1686) showed hopeful results, with activity against T790M mutations and sparing wild-type EGFR (37–39). CO-1686, is currently in a phase I/II clinical trial in patients with EGFR mutated advanced NSCLC that have received prior EGFR-directed therapy (cfr Table 3). AZD9291 is an irreversible EGFR-TKI with activity against T790M mutations. A recent phase I study with AZD9291 showed clinical activity in patients with confirmed EGFR T790M mutation NSCLC, with durable responses of >6 months (40).

### Combination therapy

In a combinatorial approach, the aim is to inhibit the primary oncogene in combination with compensatory signaling pathways. In a preclinical trial, simultaneous targeting of MET and EGFR in tumors with MET amplification showed significant tumor regression (41). Another studied combination therapy is an EGFR-TKI with the EGFR monoclonal antibody cetuximab. In a phase I/II trial of cetuximab and afatinib, objective response was observed in 40% of the patients with acquired resistance to erlotinib or gefitinib (42). A phase I/II trial of cetuximab and erlotinib in patients with lung adenocarcinoma and acquired resistance to erlotinib, showed no significant activity (43).

Another approach is the inhibition of the heat shock protein 90 (Hsp90). Hsp90 belongs to a family of proteins called molecular chaperones that are involved in the stabilization and folding of many signaling proteins (collectively referred to as Hsp90 “clients”) that are dysregulated in cancers because this protein is needed for proper folding of oncogenic kinases. EGFR is one of the most potent oncogenic client proteins of Hsp90. Several preclinical trials showed good responses in T790M mutation TKI-resistant model, when a TKI is combined with a Hsp90-inhibitor (44, 45). Phase I and II trials are ongoing (Table 3).

### Practical management

A recent review of Cadranel et al. (1) suggests a practical guideline for patients with an acquired EGFR-TKI resistance. For patients with primary resistance no guideline was provided due to lack of evidence-based data.

Progression is defined by mono- or multisite progression or by rapid or a slow progression. The choice of therapy depends on the type of progression.

Rapid progression is most likely induced by resistance mechanisms independent of EGFR signaling pathway or transformation to small-cell cancer, in contrast with slow progression that is associated with EGFR dependent resistance mechanisms. Performing a re-biopsy is interesting to understand the underlying mechanism and can guide through the therapeutic options.

For rapidly progressive disease or multi-metastatic disease, a distinction between transformation to SCLC and non-EGFR dependent resistance is made. Afterwards the appropriate chemotherapy or therapeutic trial is selected. For slowly progressive or oligo-metastatic disease, chemotherapy alone is suggested or TKI with or without loco-regional treatment, with or without chemotherapy.

## Conclusion

In the reported cases, patients with EGFR mutation positive metastatic NSCLC developed locally progressive disease at the site of the primary tumor after upfront treatment with a TKI for, respectively, 18, 26, and 40 months.

All three patients were treated with local radiotherapy with curative dose intent. TKI was temporarily stopped and restarted after radiotherapy. With this local treatment good disease control was achieved in all of the patients.

In the first patient, an ongoing disease stabilization is observed. In the second case, progression was seen 11 months after local therapy with a pleural effusion, proven to be adenocarcinoma with EGFR mutation. A switch to systemic chemotherapy was made, which is supported by the recently presented results of the important IMPRESS trial. Four months later brain metastasis were diagnosed which where treated with whole brain radiotherapy (5 Gy × 4 Gy). Erlotinib was continued afterwards.

The third patient is still without disease progression.

The treatment approach for all the patients is in accordance with the proposed treatment options in the literature. One can discuss about the choice between surgery and radiotherapy in cases with locally progressive disease. In our cases, radiotherapy was chosen because it is less invasive. Although surgery can offer an opportunity to obtain new tissue for molecular analysis.

In summary, there are different options for treatment beyond progression in EGFR mutation positive metastatic NSCLC. The optimal strategy is still to be defined, but will most probably remain subject to a personalized approach. Our examples showed that the combination with local high-dose radiotherapy is feasible and may be appropriate to control a single site of progression.

## Conflict of Interest Statement

The authors declare that the research was conducted in the absence of any commercial or financial relationships that could be construed as a potential conflict of interest.
